# Subacute Assessment of the Toxicity and Antidepressant-Like Effects of *Origanum Majorana* L. Polyphenols in Swiss Albino Mice

**DOI:** 10.3390/molecules25235653

**Published:** 2020-11-30

**Authors:** Amal Amaghnouje, Hamza Mechchate, Imane Es-safi, Smahane Boukhira, Ali S. Aliqahtani, Omar M. Noman, Fahd A. Nasr, Raffaele Conte, Anna Calarco, Dalila Bousta

**Affiliations:** 1 Laboratory of Biotechnology, Environment, Agrifood, and Health (LBEAS), University of Sidi Mohamed Ben Abdellah, Faculty of Sciences, University Sidi Mohammed Ben Abdellah, BP 1796-ATLAS Fes, Morocco; Amal.amaghnouje@usmba.ac.ma (A.A.); imane.essafi1@usmba.ac.ma (I.E.-s.); smahaneboukhira@gmail.com (S.B.); Dalila.bousta@usmba.ac.ma (D.B.); 2 Department of Pharmacognosy, College of Pharmacy, King Saud University, P.O. Box 2457, Riyadh 11451, Saudi Arabia; alalqahtani@ksu.edu.sa (A.S.A.); onoman@ksu.edu.sa (O.M.N.); fnasr@ksu.edu.sa (F.A.N.); 3 Research Institute on Terrestrial Ecosystems (IRET)—CNR, Via Pietro Castellino 111, 80131 Naples, Italy; Raffaele.conte86@tiscali.it (R.C.); anna.calarco@cnr.it (A.C.)

**Keywords:** *Origanum majorana* L., polyphenols, antidepressant-like, forced swimming test, tail suspension test, OECD 407

## Abstract

*Origanum majorana* L. is a plant commonly used in folk medicine to treat depression and several neurological disorders. This study aims to evaluate the antidepressant-like effect of the *Origanum majorana* L. polyphenols (OMP) obtained from the aerial parts using two different depression model tests: The forced swimming test (FST) and the tail suspension test (TST) in Swiss albino mice. The experiments were performed on days 1, 7, 14, and 21 with daily administration of different treatments. Two different doses were chosen for this study (50 and 100 mg/kg), and paroxetine was used as a positive control. Immobility as a consequence of the depression state was significantly reduced following the treatment with OMP, indicating an antidepressant effect. A subacute toxicity study was also performed following the Organization for Economic Co-operation and Development (OECD) Guidelines (407), showing no sign of toxicity for the studied doses. The phytochemical screening revealed the presence of 12 components, all belonging to polyphenols: Arbutin, rosmarinic acid, ursolic acid, quercetin-3-*O*-glucoside, quercetin-7-*O*-glucuronic acid, luteolin-7-*O*-glucoside, kaempferol-3-0-glucuronic acid, Kaempferol-3-0-pentose, caffeic acid, catechin, quercetin, and rutin. These findings suggest that *O. majorana* has interesting antidepressant-like properties, which deserve further investigation.

## 1. Introduction

Depression is a mood disorder that causes a persistent feeling of sadness and loss of interest and can interfere with daily functioning [1]. Besides, the World Health Organization (WHO) reported that more than 300 million people currently suffer from depression [2] However, the current antidepressants available by chemical synthesis have low response rates and can have severe side effects [3]. The natural products in drug discovery and development have become the focus of attention for many researchers as they have a higher safety than synthetic drugs [4,5]. 

Several studies defended the monoamine hypothesis linking depression to an imbalance in the monoamine neurotransmitters [6]. Specifically, the reduction of norepinephrine (NE) and serotonin (5-HT) and or receptor expression enhances or leads to depression [6].

Serotonin is one of the classes of monoamine neurotransmitters produced within axon terminals. In response to an action potential, it is released and then diffuses across the synapse to activate postsynaptic receptors at the 5-HT receptor.

NE is a monoamine neurotransmitter released in stress reactions, and its role has been increasingly recognized among those stress hormones. In the brain, NE is produced primarily by noradrenergic neurons in the locus coeruleus and is released almost throughout the brain areas. It is participating in a variety of behavioral processes such as learning and memory, attention, mood, and anxiety [7].

Different types of antidepressants have been used. Side effects, such as sexual dysfunction, coronary heart disease, and fracture risk, have been reported using these antidepressants [8], one of these being is paroxetine. Many studies have associated paroxetine with several side effects [9]. These include the effects on congenital disabilities, gestational hypertension, male fertility, hyperprolactinemia, aggression, cognitive impairment in the elderly, autism, sexual side effects, weight gain, and akathisia in children and adolescents [9]. 

In the search for a novel drug product for the treatment of depressive disorders, a large number of polyphenol purified of medicinal plants such as chlorogenic acid from *Prunus domestica* [10], rosmarinic acid, and rutin from *Melissa officinalis* [11] caffeic acid from *Lavandula officinalis* L. [12] have been investigated for their therapeutic potential in a variety of animal models.

*Origanum majorana* L. polyphenols (OMP), which are natural compounds extracted from it leaves, possess several beneficial properties, such as reducing the risk of heart diseases, cancer, and oxidative stress [13]. Polyphenols are an excellent source of new remedies for such disorders.

This study was undertaken to evaluate the antidepressant activity effect of polyphenols from *O*. *majorana* using two tests: The forced swimming test (FST) and the tail suspension test (TST) on Swiss albino mice. The extract was characterized using LC-MS technique.

## 2. Results

### 2.1. Tail Suspension Test

**Figure 1:**
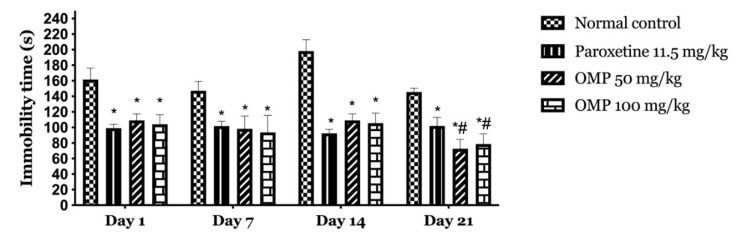
Temporal effects of *Origanum majorana* L. polyphenols (OMP) on immobility time in the tail suspension test (TST). Values are expressed as mean ± standard error of the mean (SEM). The analysis was done using two-way repeated-measures analysis of variance followed by the Tukey post-hoc test. (*) *p* < 0.001, compared with normal control; (**#**) *p* < 0.001, compared with paroxetine.

The effect of the oral administration of OMP and paroxetine at days 1, 7, 14, and 21 in TST were shown in Figure 1.

The analysis of immobility time in TST revealed significant differences, F (3, 80) = 325.1; *p* < 0.001, by treatments; days of treatment, F (3, 80) = 8.277, *p* < 0.001; and interaction between factors, F (9, 80) = 11.96, *p* < 0.001. The post hoc test showed à significant difference between all groups and normal control (*p* < 0.001), and the OMP at the different doses decreased the immobility time significantly compared to paroxetine on the 21st day.

### 2.2. Forced Swimming Test 

**Figure 2:**
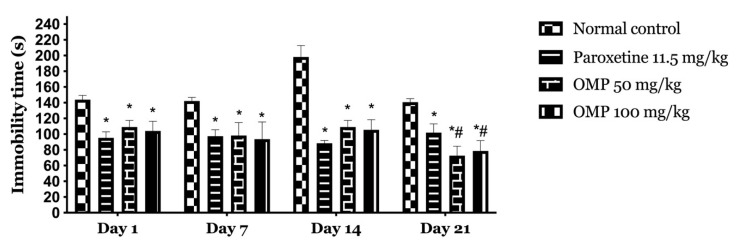
Temporal effects of OMP on immobility time in the forced swimming test (FST). Values are expressed as mean ± SEM. The analysis was done using two-way repeated-measures analysis of variance followed by the Tukey post-hoc test. (*) *p* < 0.001, compared with normal control; (**#**) *p* < 0.001, compared with paroxetine.

The effect of oral administration of OMP and paroxetine on days 1, 7, 14, and 21 in FST were shown in Figure 2. 

The analysis of immobility time in revealed significant differences, F (3, 80) = 167.7; *p* < 0.001, by treatments; days of treatment, F (3, 80) = 23.26, *p* < 0.001; and interaction between factors, F (9, 80) = 9.837, *p* < 0.001. The post hoc test showed a significant difference between all groups and normal control (*p* < 0.001) and no significant difference compared to the paroxetine on the 1st, 7th, and 14th days of the test. 

### 2.3. Toxicity Study

Oral administration of OMP for four weeks did not cause any noticeable change in the mice’s general behavior. There was no significant difference in body weight compared to the control group. Both the control and treatment groups appeared healthy during the whole study period. Administration of OMP for 28 consecutive days did not alter the biochemical parameters (Table 1). Both doses appear to be safe in accordance with the European Medicines Agency reports on the plant (EMA/HMPC/166517/2015).

Table 2 indicates the effect of the OMP on relative organ weight. According to the results, there was not a significant change in the relative weight of all organs of all doses of OMP when compared to the normal control.

### 2.4. Fraction Analysis

The injection was made in flow (FIA = flow injection analysis), so there was no chromatographic separation of the molecules. According to the molecular weight of the typical fragments, the identification was performed.

**Figure 3:**
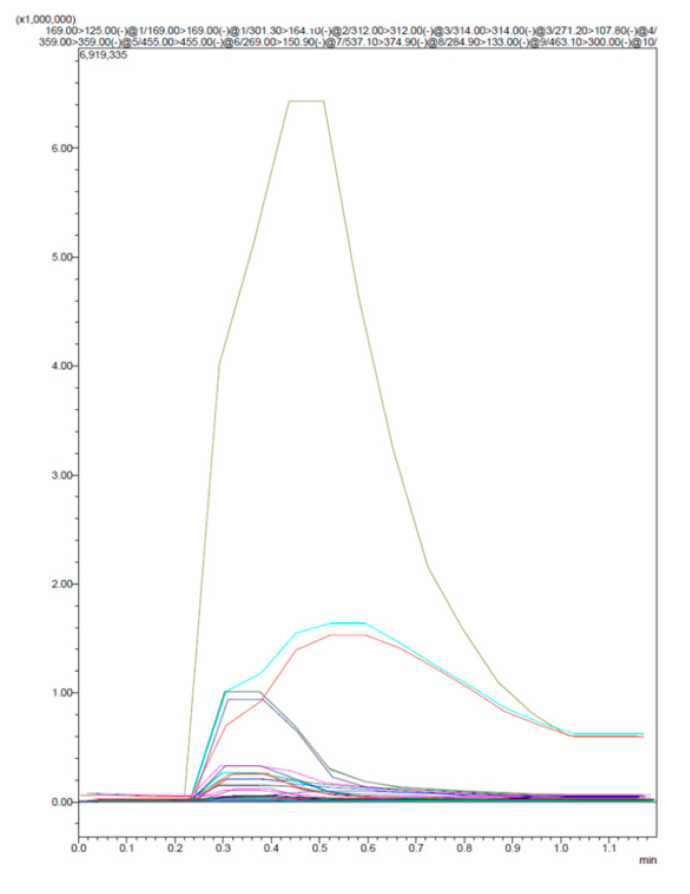
LC/MS results of OMP based on the Area Under Curve (AUC) of the molecules.

The analysis of OMP (Figure 3; Table 3) revealed the presence of 12 molecules, all belonging to the polyphenol family (arbutin, rosmarinic acid, ursolic acid, quercetin-3-*O*-glucoside, quercetin-7-*O*-glucuronic acid, luteolin-7-*O*-glucoside, kaempferol-3-0-glucuronic acid, Kaempferol-3-0-pentose, caffeic acid, catechin, quercetin, and rutin) confirming so by the accuracy of the extraction method.

## 3. Discussion

The FST and TST are two validated models used to assess putative antidepressant compounds. Immobility time in these two tests is an index for measuring antidepressant-like activity. In the present work, the OMP was administered orally. This is the common route of medication in psychiatric patients. For 21 days, it significantly reduced immobility time in both the TST and FST, suggesting that OMP has antidepressant-like effects in mice from the first day.

FST and TST tests are sensitive and relatively specific to all depression treatments, including tricyclic antidepressants (TCAs), selective serotonin reuptake inhibitors (SSRIs), monoamine oxidase inhibitors (MAOIs), and atypical antipsychotics [14]. In FST, mice are forced to swim in a limited space from which they cannot escape and, after periods of agitation, they stop their attempts to escape and become immobile. The characteristic behavior scored in this test is termed as immobility and swimming. Antidepressant drugs can reduce the immobility time, increase swimming behavior, and depend on the concentration and the type of antidepressant drug administered [15]. While on the TST, Mice are suspended by their tails and cannot escape or hold on to nearby surfaces. The resulting escape-oriented behavior is quantified during this test. The tail-suspension test is useful for screening new antidepressant treatments and testing other manipulations that are likely to influence behaviors linked with depression [16].

The TST shares with FST the potential to induce an immobility behavior, sometimes referred to as despair, in mice, which is claimed to mimic a condition similar to human depression [17]. It has been argued that the TST is less stressful than FST and has greater pharmacological sensitivity [18].

The LC/MS-MS revealed the presence of polyphenols known to have an antidepressant effect. Rosmarinic acid and caffeic acid may produce antidepressive-like activity in the FST test via some mechanisms, including monoamine transporters and inhibition of monoamine oxidase [19]. It is known that the regulation of hippocampal neurogenesis is associated with the pathogenesis of depression. Some studies have reported the neurogenesis effect of rosmarinic acid and caffeic acid on newborn cells in the dentate gyrus of the mouse hippocampus [20]. Therefore, neurogenesis may explain the decrease in immobility time in the 21st days in the groups treated with OMP compared to paroxetine. Quercetin is a polyphenol with multiple biological activities, including the antidepressant effect, it could shorten mice’s immobility time in the FST and TST [21,22]. Quercetin could alleviate LPS-induced depression-like behaviors and impairment of learning and memory, the mechanism of which might be involved with regulating the Brain-derived neurotrophic factor (BDNF)-related imbalance expression of Copine 6 and TREM1/2 in the hippocampus and the prefrontal cortex (PFC) [21].

Ursolic acid in the TST indicated an antidepressant-like effect. This effect is likely mediated by an interaction with the dopaminergic system through the activation of dopamine D(1) and D(2) receptors [23]. Other molecules were also reported to have an antidepressant-like effect like Kaempferol [24], rutin [25], and Luteolin [26] explaining by so the overall activity of the extract.

## 4. Materials and Methods

### 4.1. Plant

The leaves of Marjoram (*O. majorana* L.) were collected from Rissani in the south of Morocco. The plant was identified and authenticated by Professor A. Bari (botanist, Laboratory of Biotechnology and Conservation of Natural Resources. Faculty of Science Dhar El Mahraz, Fez). A voucher specimen (DACB: BPRN74) has been deposited in the Herbarium of the LBEAS laboratory for further reference.

### 4.2. Polyphenol Extraction

With 150 mL of methanol, we extracted 50 g of *O. majorana* L. three times at 40 °C for 3 h (maceration). The extract was concentrated and dissolved in 250 mL of water, washed after that three times with 100 mL hexane and three times with 100 mL chloroform to eliminate the chlorophyll and the caffeine. The aqueous phase was extracted three times, with 100 mL ethyl acetate. The ethyl acetate was evaporated under reduced pressure and the residue re-dissolved in 150 mL water freeze-dried to obtain the polyphenol extract [27].

### 4.3. Animals

Swiss albino mice weighing approximately 25–30 g used for the tests. They were housed in polypropylene cages in an air-conditioned room, with the temperature maintained at 24 ± 3 °C. The mice were provided with a nutritionally adequate diet and drinking water. 

Mice were randomly assigned to treatment groups (*n* = 7–8/treatment): (50 and 100 mg/kg OMP, and paroxetine at 11.5 mg/kg). Animals received daily treatments for 21 days and were tested one hour after the final dose. Animals were transferred from their cages to the experimental room and allowed to habituate for at least one hour before beginning the behavioral tests. Testing was performed in a randomized order during the animal’s active period (6 a.m.–6 p.m.). Experiment procedures were carried out in accordance with the international guidelines for the care and use of laboratory animals [28], and the protocol was approved by the institutional animal ethical committee (12/2018/LBEAS). The treatments were given orally to the animals by intragastric gavage using a stainless steel bulb tipped gavage needle in order to deliver it into the stomach.

### 4.4. Behavioral Tests

#### 4.4.1. Forced Swimming Test

The FST was initially described by [29] and is the most widely used pharmacological model for assessing antidepressant activity [30]. The animal is maintained in a rectangular pool (50 × 30 × 60 cm), filled to a 25 cm depth with water at room temperature at 22–25 °C [31]. The test assumes that the animal will swim actively in order to escape from stressful stimuli. The total duration of immobility was recorded during 4 min of the whole test duration of 6 min [32].

#### 4.4.2. Tail Suspension Test

For 6 min, the animal was hung by the tail 2 cm from the end in a box of dimensions 50 × 25 × 50 cm, the head preserved at 15 cm above the bottom of the box. Data was recorded only in the last 4 min of the test. Immobile was scored as a failure to make any struggling movements. The animal was considered immobile only when they hung passively and completely motionless [32].

### 4.5. Fraction Analysis 

Qualitative profiling of the OMP was made by ultra-high-performance liquid chromatography with triple quadruple mass spectrometry. In particular, the analysis was performed on a Shimadzu Ultra-High-Performance Liquid Chromatography (Nexera XR LC 40), coupled to an MS/MS detector (LCMS 8060, Shimadzu Italy, Milan, Italy). The MS/MS was operated with electrospray ionization (ESI) and controlled by Lab Solution software, which simultaneously provided quick switching from low energy scan at 4V (full scan MS) to high energy scan (10–60 V ramping) during a single LC run. The low-CE experiments provided information about the intact molecular ion (e.g., M^+^, [M + H]^+^), while the high-CE scan generates fragment ion information. The source parameters were set as follows: nebulizing gas flow 2.9 L/min, heating gas flow 10 L/min, interface temperature 300 °C, DL temperature 250 °C, Heat block temperature 400 °C, drying gas flow 10 L/min. Qualitative analysis was performed developing an in-house database comprising the secondary metabolites of polyphenols. Standards for each phenolic compound were identified according to their chromatographic separation on a Phenomenex Kinetex polar C18 column (3 × 100mm, 2.6 μm, Phenomenex, Torrance, CA, USA) with mobile phase consisting of acetonitrile: water + 0.01% formic acid (5:95, *v*/*v*; isocratic). Chromatography separation was confirmed by mass identification based on the precursor ion’s detection and at least one characteristic fragment ion. (detailed in the supplementary file).

OMP extracts were suspended in 2 mL of solution water: Acetonitrile 1:1. 20 microliters of these solutions were diluted in 980 microliters of acetonitrile and injected for LC-MS/MS analysis. 

Polyphenols identification was performed by comparison with retention times of database compounds and confirmed by their characteristic fragmentations obtained in flow injection with a mobile phase consisting of acetonitrile: water + 0.01% formic acid (5:95, *v*/*v*).

### 4.6. Toxicity 

#### 4.6.1. Subacute Toxicity

The oral toxicity study of OMP was performed according to the Organization for Economic Co-operation and Development (OECD) guideline 407. Mice of both sexes were assigned randomly to three groups (*n* = 5/group). Groups II–III received 50 and 100 mg/kg of the OMP, respectively. While the group I received NaCl 0.9% (5 mL/kg) only, for 28 days. The weights of all animals were measured every day.

On the last day, the animals were sacrificed after anesthesia, and blood collection was performed. The liver, lungs, kidneys, spleen, and adrenal glands were excised, weighed, and assessed macroscopically. The relative organ weight of each harvested organ was calculated using the relation:

Relative organ weight (%) = (organ weight/body weight) × 100%

The euthanization was done following the American Veterinary Medical Association (AVMA) guidelines for the euthanasia of animals.

#### 4.6.2. Serum Biochemical Analysis

Blood for biochemical analysis was gently placed in plain bottles to avoid hemolysis of the blood cells. In the next step, blood serum was obtained by centrifugation of the blood sample to analyze the biochemical parameters (aspartate aminotransferase (AST), alanine aminotransferase (ALT), urea, and creatinine in the serum).

### 4.7. Statistical Analysis

Experimental data were presented as mean ± SEM. Significant differences were compared by two-way ANOVA (Tukey post hoc test for behavioral test and Dunnett’s test for the toxicity test). The statistical difference was regarded as *p* < 0.05.

## 5. Conclusions

The results obtained in this study suggest that the polyphenols fraction of *O. Majorana* possesses remarkable antidepressant effect at doses of 50 and 100 mg/kg. Even better than the positive control (paroxetine). The fraction was relatively non-toxic in mice. Hence it is possibly safe for human consumption. This is the first investigation of OMP on depression. Other studies are now necessary to discover the mechanism of action, and further work is required to identify the active components with antidepressant-like activity that might be useful in the prevention and therapy of mood disorders.

## Figures and Tables

**Table 1:** Effect of subacute administration of OMP on biochemical parameters in male mice (*n* = 5).

	Normal Control	OMP 50 mg/kg	OMP 100 mg/kg
Uree (g/L)	0.28 ± 0.02	0.26 ± 0.08	0.23 ±0.01
Creatinine (mg/L)	3.40 ± 0.31	3.333 ± 0.89	4 ± 0
ALT (U/L)	45.80 ± 1.11	37 ± 5.17	31 ± 6.21
AST (U/L)	307.7 ± 30.37	265 ± 22.1	322 ± 29.1

Values are expressed as mea1n ± SEM.

**Table 2:** Effect of subacute administration of OMP on the relative weight of organs.

	Liver (g)	Kidneys (g)	Spleen (g)	Adrenal Gland (g)	Lungs (g)
Normal control	8.95 ± 0.52	1.79 ± 0.18	0.74 ± 0.15	0.24 ± 0.01	0.88 ± 0.28
OMP 100 mg/kg	7.749 ± 0.21	1.74 ± 0.06	0.67 ± 0.04	0.21 ± 0.11	0.80 ± 0.09
OMP 50 mg/kg	7.84 ± 0.16	1.82 ± 0.12	0.8 ± 0.11	0.17 ± 0.08	0.91 ± 0.3

Values are expressed as mean ± SEM.

**Table 3:** LC/MS-MS identified composition of OMP.

Molecule	Fragment Analyzed	AUC
Arbutin	271.20 > 107.80	2,295,593
Rosmarinic acid	359.00 > 359.00	113,966,325
Ursolic acid	455.00 > 455.00	2,400,530
Quercetin-3-*O*-glucoside	463.10 > 300.00	419,677
Quercetin-7-*O*-glucuronic acid	477.00 > 301.00	1,090,191
Luteolin-7-*O*-glucoside	447.10 > 285.00	4,024,661
Kaempferol-3-0-glucuronic acid	461.10 > 284.00	34,807,891
Kaempferol-3-0-pentose	417.10 > 284.00	661,262
Caffeic acid	179.00 > 135.00	2,334,092
Catechin	289.00 > 245.00	515,400
Quercetin	301.00 >151.00	16,011,331
Rutin	609.00 > 301.00	35,325

## Supplementary Materials

The following are available online, LC/MS results of OMP based on the AUC of the molecules in both flow injection and on-column separation.
