# Supplementary material for: Subacute Assessment of the Toxicity and Antidepressant-Like Effects of Origanum Majorana L. Polyphenols in Swiss Albino Mice
Source: Molecules. 2020 Nov 30;25(23):5653. doi: 10.3390/molecules25235653 (PMC7730305; doi:10.3390/molecules25235653)
Supplement: Supplementary file 1 [file molecules-25-05653-s001.pdf]

Molecule	Retention Time	OMP extract	Standard in ACN (dil 1:1000)
Arbutin	2 min	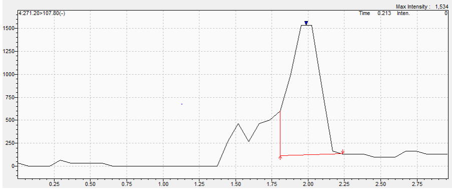	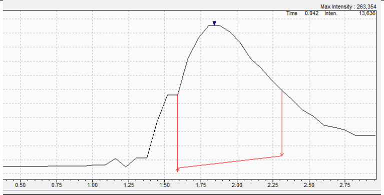
Rosmarinic acid	1,48 min	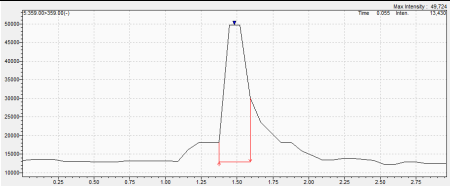	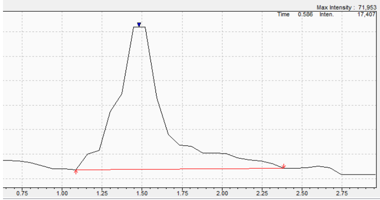
Ursolic acid	1,50 min	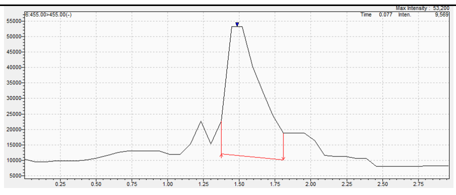	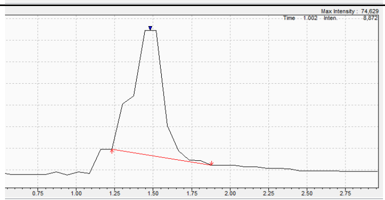
Quercetin-3-O-glucoside	1,49 min	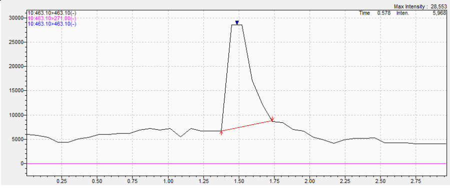	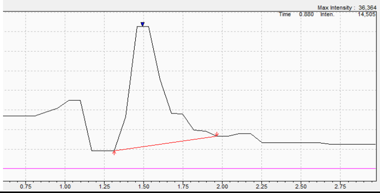
Quercetin-7-O-glucuronic acid	1,49 min	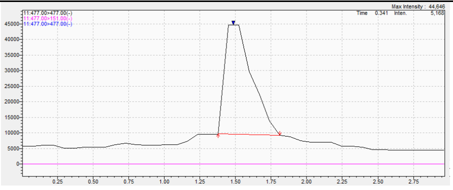	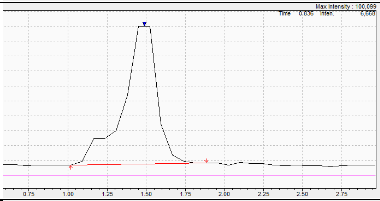
Luteolin-7-O-glucoside	1,52 min	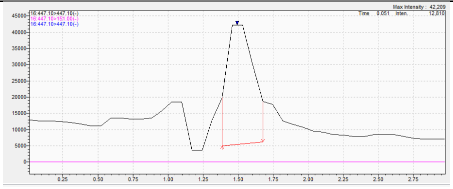	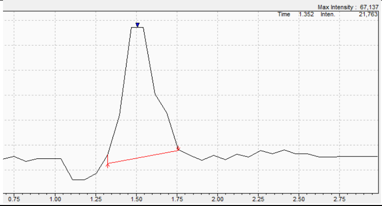
Kaempferol-3-o-glucuronic acid	1,48 min	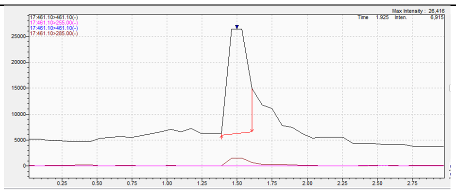	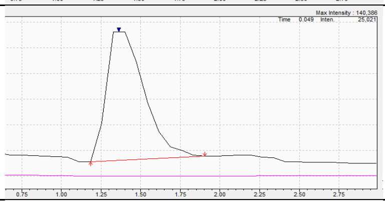
Kaempferol-3-o-pentose	1,51 min	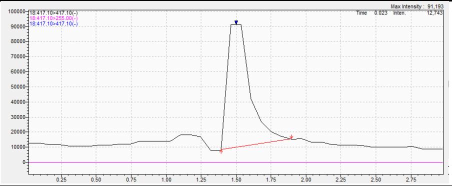	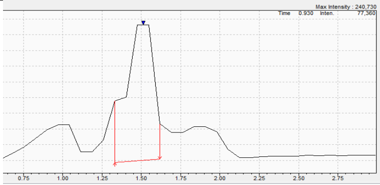
Caffeic acid	1,85 min	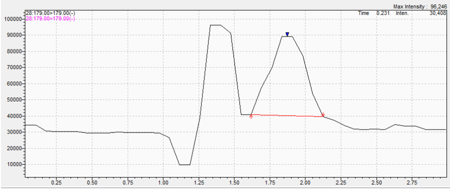	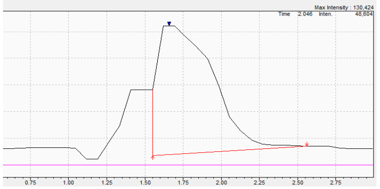

Catechin	1,35 min	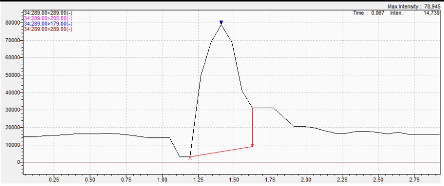	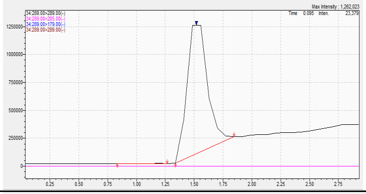
Quercetin	1,4 min	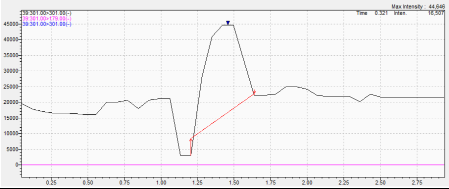	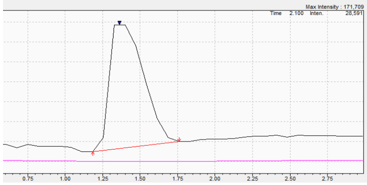
Rutin	1,45 min	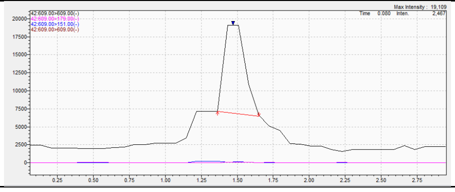	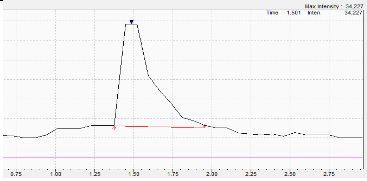
